# Variations in the evaluation of the Edinburgh Postnatal Depression Scale in different psychotic states and language versions

**DOI:** 10.1192/j.eurpsy.2025.2418

**Published:** 2025-08-26

**Authors:** N. Igor, B. Larisa

**Affiliations:** 1 Department of Mental Health, Medical Psychology, and Psychotherapy, State University of Medicine and Pharmacy Nicolae Testemițanu, Chisinau, Moldova, Republic of

## Abstract

**Introduction:**

Patients with postpartum depression may have an increased risk of developing schizophrenia or bipolar affective psychosis. Conversely, women with schizophrenia may have a higher risk of developing depression during the perinatal period, which could be screened using the EPDS. The presence of cutoff effects complicates the statistical analysis of the Edinburgh Postnatal Depression Scale (EPDS). Sensitivity and specificity, two key parameters, cannot be treated as independent variables in such cases. To improve the precision of EPDS evaluations across different populations, versions with multiple cutoff points are preferable. While linear regression correlations may produce statistically significant results, they can be misleading when nonlinear relationships are present. In these instances, alternative statistical methods are recommended, such as hierarchical (Bayesian) models, bivariate (random effects) models assuming normal distribution, and joint modeling of sensitivity and specificity. Additionally, models with multiple cutoff values for sensitivity and specificity are particularly useful.

**Objectives:**

This study aims to review the literature and assess the validity of the EPDS in its various language versions and across different psychotic states.

**Methods:**

The literature data on the EPDS, across different language versions and psychotic states, was statistically analyzed, focusing on specificity, sensitivity, and the influence of cutoff thresholds.

**Results:**

The analysis of 42 pairs of sensitivity and specificity data using linear regression showed a statistically significant moderate negative correlation (Pearson’s r =−0.4342, p = 0.0018). Visual aids such as the histogram and Q-Q plot indicated the absence of normal distribution, confirmed by formal normality tests. The normality test results were as follows: Kolmogorov-Smirnov: D(84) = 0.135, p<0.001; Shapiro-Wilk: W = 0.817, p<0.001.

**Conclusions:**

The Kruskal–Wallis one-way ANOVA test revealed statistically significant differences among the three variables (sensitivity, specificity, and cutoff threshold), with a test statistic of H(2) = 79.647, p<0.001. The Receiver Operating Characteristic (ROC) curve is widely regarded as the most reliable tool for representing the relationship between sensitivity, specificity, and cutoff thresholds across different language versions of the EPDS. These variations account for cultural and national characteristics, which play a significant role in the scale’s overall validity.

**Disclosure of Interest:**

None Declared

